# History of Three Cases of Intermittent Fever, Showing the Natural Course of the Disease

**Published:** 1856-10

**Authors:** J. Forsyth Meigs


### MEDICAL EXAMINER.

NEW SERIES.—NO. XLII.—OCTOBER, 1856.

---

### ORIGINAL COMMUNICATIONS.

---

*History of three cases of Intermittent Fever, showing the natural course of the disease.* By J. Forsyth Meigs, M. D.

The natural history of disease, or, in other words, the characters of disease uninfluenced by human interference, must always be a subject of the greatest interest to all but the merely routine practitioner. And yet the treatment of most important diseases is, at this time, so well established by general consent among physicians, that there are few opportunities of witnessing the successive phenomena of the morbid state excepting as more or less disturbed or controlled by therapeutical intervention. Especially is this true of the periodical fevers, the treatment of which is so clearly and explicitly formulated by the best authorities in medicine, that it is altogether doubtful whether a regular practitioner has any right to leave a case to the powers of nature alone. The following cases of intermittent fever occurred to me after I had been several years in practice, and were the first in which I saw the unchecked tendencies of the disease,—tendencies which showed me how comparatively impotent must have been the medical art in a contest with such a malady before the discovery of cinchona. It may be said by some that even these cases cannot be properly considered as having been left to nature's own management, since they were treated at first by the homœpathic method. I have not, however, the slightest hesitation in so considering them, believing that the influence of the infinitesimal dosing to which they had been subjected, was, as to any real effect, equivalent to nothing. They interested me very much at the time they occurred, as proving to demonstration the manifest and most beneficent control exercised by a remedy over a disease, an effect which I had not doubted to be sure before, but which, nevertheless, was now exhibited to me in the most delightful manner.

The cases are as follows: Two sisters, one 16 years of age, the other 25, spent a part of August, 1851, at Gloucester, New Jersey. They came home in that month, and remained well through the following fall and winter. On the 5th of May, 1852, the elder sister was taken sick with tertian ague which continued during two weeks. At the end of the second week the disease assumed the quotidian type, and so continued, without the slightest break, up to the 16th of June, just six weeks from the onset. The chills occurred sometimes at midday, but generally, indeed almost always, between 3½ and 4 P. M. They lasted one or two hours usually, were violent and attended with shaking and with chattering teeth. Then came a "raging" fever, and after a few hours, a copious, wringing sweat. The patient slept very badly at night, had violent headaches, was without appetite, and vomited nearly every afternoon when the chill came on. She mentioned one curious fact, to wit: that when she lay on her right side, she rarely vomited, but instantly on turning to the left side, would vomit violently. She usually rejected large quantities of green bile.

During the first six weeks of her illness she had been frequently visited by a homœopath, who had dosed her with various infinitesimals. When I saw her on the 16th of June she was as white as chalk; her lips were white, and the mucous membrane of the mouth pale; her hands and ears were transparent; (she was naturally a healthy and hearty subject both in appearance and in fact;) the impulse of the heart was strong, jerking, and sudden, the cardiac sounds were metallic in tone and attended with a soft bruit; she was so weak that she could not stand up without giddiness, sickness, and disposition to faint. She ate almost nothing, and was as much broken down in spirits as in strength, crying constantly, as she told me, in her bed, and entreating her mother to give up the infinitesimals and try some more potent means of cure.

My first visit was at 8 P. M., the patient having then the fever which followed her daily chill. It was too late to give quinine that evening, but on the following morning she began it, and continued to take it through that day. She had her chill and fever that day in spite of the remedy; but the following day, after taking quinine in the morning, she it missed entirely. She was now put upon the use of milk punch, small doses of quinine, iron, porter, meat, and general good diet, and rapidly recovered her usual health.

The other sister was not attended by myself, for, a few days before I was called in, after having suffered from the disease during five weeks, though less severely than my patient, she had obtained from an apothecary store some one of the usual prescriptions for intermittent fever, and was cured by it in a very short time.

The third case was one that occurred to me some years since. The subject was a boy ten years old, who had been spending the latter part of the summer on the Chesapeake Bay. Soon after his return to this city, he was attacked with intermittent fever. His mother sent for a homœopathic physician, who visited him daily and prescribed. During the first six days, the attack was of the tertian form. It then changed to a quotidian, and so continued for six days longer. On the eleventh day there appeared some hæmorrhage from the bowels, which, with other alarming symptoms, and the continuation of the paroxysms, so terrified the mother, that, on the morning of the thirteenth day, she dismissed the homœopath and sent for me. The child was much prostrated and looked ill. He had already become very thin, and was entirely without appetite. The abdomen was largely distended from two causes,—the presence of gas in the intestines, and enlargement of the spleen. The latter organ was so much increased in size as to extend nearly to the umbilicus. There was no cough nor any other sign of trouble in the thoracic organs, nor were there any important cerebral symptoms. The daily paroxysms of the disease were severe, lasting from about 10½ or 11 A. M. to the latter part of the afternoon.

The treatment directed was small doses of sugar of lead and opium through the day, laudanum injections at night, and a diet of farinaceous articles and light broths for the intestinal hæmorrhage; whilst quinine was given daily after the cessation of the paroxysm and prior to the attack in the morning. The quinine was given after the paroxysm which occurred on the day that I first saw the patient, and on the following day. Notwithstanding this, a paroxysm occurred on the day after my first visit (14th), but none afterwards. The remedy broke up the paroxysmal character of the disease from that moment, and the hæmorrhage ceased on the 14th day. The spleen returned rapidly to its normal size, and, with the exception of some troublesome constipation and a prostration which was slow in passing away, the patient had no further difficulty.

These cases cannot but be, it seems to me, interesting to the physician. Though they demonstrate only what we already know, the efficacy of quinine in the treatment of malarial disease, they do it in so plain and conspicuous a manner as to give to this truth a freshness which is delightful in these days of isms and quackeries, when doubt and skepticism as to the utility of the medical art are only too common. The history of the first case is particularly interesting in showing, not only the efficacy of the remedy, but also the effects of the disease, when left to its natural unchecked development, upon the constitution. We may, in reflecting upon the wretched condition of exhaustion to which the patient had been reduced, in considering the suffering she each day went through with,—the chill, the vomiting, the fever, with its aches and pains, the sweat, with its wretched prostration of soul as well as body, the misery of daily anticipation, and the sure progession of the daily weakness, learn to value the greatness of the boon conferred upon us by Providence in the discovery of the Peruvian bark.

Habit and routine familiarize us to everything, and physicians themselves are, perhaps, less thankful than they should be for the possession of a means of treatment the value of which is priceless; while the ungrateful people, who often cry out against quinine as a dangerous remedy, should learn to estimate more accurately its true preciousness.

